# Hypermagnesemia in a Cat Secondary to Suspected Intoxication

**DOI:** 10.1111/vec.70021

**Published:** 2025-08-30

**Authors:** Jackson A. Griffith, Melissa R. Santonocito, Katie D. Mauro

**Affiliations:** ^1^ Michigan State University Veterinary Teaching Hospital East Lansing Michigan USA

**Keywords:** acute kidney injury, feline, magnesium

## Abstract

**Objective:**

To describe the management of a unique case of hypermagnesemia in a cat.

**Case Summary:**

An 8‐year‐old spayed female domestic shorthair cat was presented for acute‐onset stupor, hypothermia, and bradycardia. Overtly elevated ionized (>1.5 mmol/L [>3.6 mg/dL]; reference interval: 0.4–0.8 mmol/L [0.9–1.9 mg/dL]) and total magnesium (3.0 mmol/L [7.3 mg/dL]; reference interval: 0.74–1.0 mmol/L [1.8–2.5 mg/dL]) concentrations were identified. A thorough history uncovered the long‐term at‐home administration of an anxiolytic supplement containing magnesium stearate as an inactive ingredient. No other sources of nondietary magnesium were identified. A diagnosis of magnesium toxicosis was reached. Secondarily, an acute kidney injury developed. After 10 days of hospitalization, the cat was successfully discharged with normalized kidney and electrolyte values.

**New or Unique Information Provided:**

Few case reports regarding magnesium toxicosis and its management exist in the veterinary literature. A rare case of hypermagnesemia is presented in this report, highlighting the importance of a complete laboratory workup that includes an evaluation of magnesium levels in previously healthy animals that present with otherwise unexplained clinical signs.

**Abbreviations**
AKI: acute kidney injury
DCT: distal convoluted tubule
FDA: Food and Drug Administration
IRIS: International Renal Interest Society
LRS: lactated Ringer's solution
RI: reference interval

## 1 Introduction

Most of the magnesium in the body is found within osteocytes and other cells [1, 2]. Serum magnesium exists mostly in an ionized (55%–65%) form, which is the physiologically active state. The second largest fraction of magnesium is protein bound (30%–40%) and is largely bound to albumin [1]. Magnesium's movement depends on electrochemical gradients and hormonal control within the intestinal and renal epithelium [1]. The primary mediators of magnesium uptake are vitamin D, aldosterone, parathyroid hormone, and prostaglandin E2, all of which operate through currently undescribed mechanisms [1]. The absorption of magnesium occurs in all segments of the small intestines and colon but is not tightly regulated. In contrast, there is strict hormonal control of magnesium reabsorption at the level of the kidneys [1, 3], with the largest proportion reabsorbed in the thick ascending loop of Henle (80%) and most regulatory mechanisms occurring within the distal convoluted tubule (DCT) [2]. Reabsorption of magnesium in the DCT depends to a degree on electrochemical gradients and competitive ion channel use of calcium [1, 2].

Magnesium functions in the cells to regulate aerobic metabolism, serving as a cofactor to sodium–potassium ATPase during oxidative phosphorylation [1, 3]. Within the immune system, nervous system, and intracellular functions such as DNA synthesis and the prevention of apoptosis, magnesium plays various roles, including influencing the movement and functions of calcium by competing as a bivalent cation [1, 3]. Most importantly for this report, magnesium is a significant cofactor for the vascular smooth muscle contraction that determines vasomotor tone [1, 3].

Hypomagnesemia is more frequently reported than its counterpart, hypermagnesemia. Previously reported causes of hypermagnesemia are limited to decreased renal clearance, neoplasia, and iatrogenic administration [1, 2, 3, 4]. Hypermagnesemia results in dose‐dependent intoxication. Initial signs affect the gastrointestinal tract, followed by hypotension, bradycardia, and progressive neural depression [1, 3, 5]. Research into the benefits of magnesium supplementation in critically ill patients and the effect of these derangements has been a topic of interest in human medicine in recent years and is becoming more prevalent in veterinary medicine [3, 5, 6]. After an extensive review of this patient's history, the only possible source of nondietary magnesium was an anxiolytic supplement containing the inactive ingredient magnesium stearate.

## 2 Case Report

An 8‐year‐old spayed female domestic shorthair cat, mainly kept indoors, was presented to the Michigan State University Veterinary Teaching Hospital as a referral for altered level of consciousness and bradycardia. Two adult cats initially lived in the household, and 6 months before presentation of this case, a third cat was adopted. Since that time, the two original cats were each offered approximately one‐half the contents of a 75‐mg capsule of vetoquinol^a^ daily. Vetoquinol, a supplement for anxiety, contains the active ingredient alpha‐casozepine as well as magnesium stearate as an inactive ingredient. The clients cannot be certain which cat(s) are ingesting the capsule contents, so it is possible that the patient has ingested as much as the whole vetoquinol capsule daily for an extended period of time. Also included in the cat's medical history was an unspecified chronic enteropathy that had been treated intermittently with metronidazole^b^.

Five days before referral, the cat developed anorexia and vomiting; 2 days later, the cat was presented to an emergency clinic. Bloodwork revealed an inflammatory leukogram, normal kidney values (BUN 11.5 µmol/L [32.3 mg/dL], reference interval [RI]: 5.4–11.4 µmol/L [15–32.0 mg/dL]; creatinine 90.8 µmol/L [1.0 mg/dL], RI: 72.6–163.5 µmol/L [0.8–1.8 mg/dL]), and a serum magnesium concentration of 1.1 mmol/L (2.7 mg/dL, RI: 0.74–1.1 mmol/L [1.8–2.7 mg/dL]). The evaluation was otherwise unremarkable, and 30 mL/kg of lactated Ringer's solution^c^ (LRS) and 1 mg/kg of maropitant^d^ were administered subcutaneously. Two days before the cat's referral to our hospital, clinical signs persisted. The primary veterinarian administered an unknown dose of cefovecin^e^ subcutaneously and prescribed oral metronidazole^b^; however, the owner was unable to administer the oral medication due to the cat's persistent anorexia. One day before referral to our hospital, the cat acutely declined to the point of lateral recumbency and fecal incontinence.

On presentation to our hospital, the cat was stuporous with a blood pressure too low to detect on Doppler, moderately bradycardic (140/min), hypothermic at 33.5°C (92.3°F), and estimated to be 10% dehydrated. A full neurologic examination was not conducted due to the critical status at presentation, but no cranial nerve abnormalities were noted. The remainder of the physical examination at presentation was unremarkable.

Initial stabilization included a 20 mL/kg bolus of LRS and active heat support. Blood pressure, measured by Doppler, was volume responsive and increased from an unreadable level to 85 mm Hg. Throughout Day 1 of hospitalization, the cat's temperature, blood pressure, and heart rate normalized; LRS (6 mL/kg/h) was provided throughout the day, but no improvement in mentation was noted. The medications administered included maropitant^d^ (1 mg/kg, IV) every 24 h throughout hospitalization to control vomiting and two doses of furosemide^f^ (1 mg/kg, IV) 12 h apart to induce diuresis and promote renal excretion of magnesium. Treatment with calcium gluconate^g^ to address hypermagnesemia was considered but not pursued. In addition, an increased serum phosphorus concentration (Table 1) raised concerns about potential soft tissue mineral deposition if calcium therapy were to be implemented.

**TABLE 1:** Serum biochemistry findings throughout the hospitalization of an 8‐year‐old cat treated for neurologic and hemodynamic instability secondary to severe magnesium toxicosis.

Analyte (serum concentration)	Day 1	Day 3	Day 5	Day 10	Reference interval
Calcium	1.6 mmol/L (6.5 mg/dL)	1.9 mmol/L (7.4 mg/dL)	1.8 mmol/L (7.2 mg/dL)	2.2 mmol/L (8.8 mg/dL)	2.3–2.7 mmol/L (9.1–10.7 mg/dL)
Magnesium	3 mmol/L (7.3 mg/dL)	1.1 mmol/L (2.8 mg/dL)	0.78 mmol/L (1.9 mg/dL)	0.70 mmol/L (1.7 mg/dL)	0.74–1.0 mmol/L (1.8–2.5 mg/dL)
Sodium	137 mmol/L (137 mEq/L)	144 mmol/L (144 mEq/L)	155 mmol/L (155 mEq/L)	156 mmol/L (156 mEq/L)	145–155 mmol/L (145–155 mEq/L)
Chloride	92 mmol/L (92 mEq/dL)	99 mmol/L (99 mEq/dL)	116 mmol/L (116 mEq/dL)	115 mmol/L (115 mEq/dL)	110–123 mmol/L (110–123 mEq/dL)
Potassium	5.5 mmol/L (5.5 mEq/L)	3.6 mmol/L (3.6 mEq/L)	2.9 mmol/L (2.9 mEq/L)	5.1 mmol/L (5.1 mEq/L)	3.8–5.4 mmol/L (3.8–5.4 mEq/L)
Phosphorus	5.7 mmol/L (17.7 mg/dL)	4 mmol/L (12.5 mg/dL)	1.2 mmol/L (3.8 mg/dL)	1.5 mmol/L (4.6 mg/dL)	0.9–1.8 mmol/L (2.7–5.7 mg/dL)
Albumin	25 g/L (2.5 g/dL)	20 g/L (2.0 g/dL)	17 g/L (1.7 g/dL)	22 g/L (2.2 g/dL)	30–39 g/L (3.0–3.9 g/dL)
Creatinine	445 µmol/L (4.9 mg/dL)	573 µmol/L (6.3 mg/dL)	182 µmol/L (2.0 mg/dL)	118 µmol/L (1.3 mg/dL)	91–209 µmol/L (1.0–2.3 mg/dL)

**FIGURE 1:**
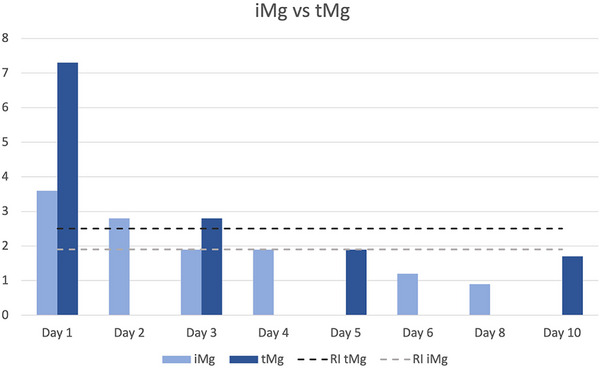
Ionized and total magnesium data throughout the hospitalization of an 8‐year‐old cat treated for neurologic and hemodynamic instability secondary to severe magnesium toxicosis. iMg, serum ionized Mg concentration; RI, upper end of reference interval; tMg serum total Mg concentration. Magnesium concentration on the Y axis, expressed in mg/dL.

A CBC performed on Day 1 of hospitalization revealed an inflammatory leukogram. Serum biochemistry panel revealed severe azotemia, International Renal Interest Society (IRIS) grade III acute kidney injury (AKI) [7], and severe electrolyte abnormalities (Table 1). A venous blood gas analysis showed ionized hypocalcemia and ionized hypermagnesemia (>1.5 mmol/L [>3.6 mg/dL], RI: 0.4–0.8 mmol/L [0.9–1.9 mg/dL]) (Figure 1). Initial lactate measurements were normal. There was a mixed acid–base disturbance on presentation characterized by severe azotemia, hyponatremia, and hypochloremia (Table 2). A urinalysis revealed a urine specific gravity of 1.020 and mild proteinuria (1+ out of 4+) with no other significant findings.

**TABLE 2:** Venous blood gas results throughout the hospitalization of an 8‐year‐old cat treated for neurologic and hemodynamic instability secondary to severe magnesium toxicosis.

Value	Day 1	Day 2	Day 3	Day 4	Day 6	Day 8	Reference interval
iMg	>1.5 mmol/L (>3.6 mg/dL)	1.2 mmol/L (2.8 mg/dL)	0.78 mmol/L (1.9 mg/dL)	0.78 mmol/L (1.9 mg/dL)	0.49 mmol/L (1.2 mg/dL)	0.37 mmol/L (0.9 mg/dL)	0.37–0.78 mmol/L (0.9–1.9 mg/dL)
iCa	1.0 mmol/L (4.0 mg/dL)	1.1 mmol/L (4.4 mg/dL)	1.1 mmol/L (4.3 mg/dL)	1.2 mmol/L (4.7 mg/dL)	1.3 mmol/L (5.2 mg/dL)	1.3 mmol/L (5.1 mg/dL)	1.3–1.5 mmol/L (5.1–6.0 mg/dL)
pH	7.31	7.26	7.42	7.50	7.36	7.38	7.26–7.4
Bicarbonate	17.2 mmol/L (17.2 mEq/L)	14.0 mmol/L (14.0 mEq/L)	27.5 mmol/L (27.5 mEq/L)	36.5 mmol/L (36.5 mEq/L)	20.6 mmol/L (20.6 mEq/L)	20.1 mmol/L (20.1 mEq/L)	13.9–22.8 mmol/L (13.9–22.8 mEq/L)
pCO_2_	4.53 kPa (34.1 mm Hg)	4.16 kPa (31.3 mm Hg)	5.61 kPa (42.2 mm Hg)	6.21 kPa (46.7 mm Hg)	4.76 kPa (35.8 mm Hg)	4.44 kPa (33.4 mm Hg)	3.62–5.51 kPa (27.2–41.4 mm Hg)
Lactate	1.7 mmol/L (15.3 mg/dL)	1.7 mmol/L (15.3 mg/dL)	0.9 mmol/L (8.1 mg/dL)	0.7 mmol/L (6.3 mg/dL)	0.5 mmol/L (4.5 mg/dL)	0.7 mmol/L (6.3 mg/dL)	1.0–4.7 mmol/L (9.0–42.3 mg/dL)

An abdominal ultrasound was performed that showed a diffusely thickened intestinal wall and jejunal lymphadenopathy, considered most consistent with the cat's reported chronic enteropathy. Changes were noted in the cat's kidneys and pancreas. Mild dilation of the right renal pelvis was noted that may have represented physiologic pyelectasia from fluid therapy or pyelonephritis. Left renal hyperechogenicity was appreciated, suggesting chronic cortical change or cortical fat. The pancreas appeared hypoechoic, which could be secondary to the presence of free fluid or represent evidence of pancreatitis. The ultrasound findings were reviewed by a board‐certified radiologist.

On Day 2 of hospitalization, a nasogastric tube was placed, and feedings were started with a renal diet^h^ at 25% of calculated daily resting energy requirement. Throughout the second day of hospitalization, the cat's blood pressure became unresponsive to fluid boluses (total 35 mL/kg LRS); therefore, norepinephrine^i^ was added for suspected vasodilatory shock. Persistent hypocalcemia (iCa 1.0 mmol/L [4.0 mg/dL], RI: 1.3–1.5 mmol/L [5.1–6.0 mg/dL]) was noted, and a constant rate infusion of calcium gluconate^g^ (5 mg/kg/h, for a total of 5 h) was initiated. Evidence of severe inflammation was still present on bloodwork; therefore, ampicillin–sulbactam^j^ (30 mg/kg, IV, q 8 h) was prescribed for possible sepsis secondary to gastrointestinal translocation.

Improvement in electrolyte and kidney values was seen over the next days of hospitalization, with a resolution of hypermagnesemia on Day 3 (Table 1). Repeat bloodwork on Day 4 showed the development of a nonregenerative anemia (HCT 19%). A blood transfusion was not administered. Norepinephrine was weaned throughout Day 4 (maximum dosage 0.5 µg/kg/min, reduced by 0.1 µg/kg/min q 4 h). The cat's heart rate, respiratory rate, and blood pressure measurements remained stable. After de‐escalation of norepinephrine, the rate of fluids was decreased to 4 mL/kg/h. The cat's voluntary appetite remained poor, but tube feedings were well tolerated and were gradually increased by 25% daily starting on Day 3, to 75% daily resting energy requirement. Subjectively, the cat's mentation became significantly brighter, and progressively more interaction was noted daily. On Days 4 through 10, supportive care was continued for ongoing gastrointestinal signs and resolving AKI. The cat's appetite returned on Day 9, and the feeding tube was removed. On Day 10, the cat was discharged from the hospital with normal kidney values (Table 1). Plans were made to follow up with the internal medicine service for further investigation of the cat's chronic enteropathy.

Three days after the cat was discharged, an evaluation was performed by the internal medicine department. Serum biochemistry panel revealed no significant abnormalities. The HCT was improved at 27% (RI: 33%–50%) with evidence of regeneration (8.8 × 10^10^/L reticulocytes [RI: 0.9–8.5 × 10^10^/L]). However, leukocytosis (43.5 × 10^9^/L [RI: 4.3–14 × 10^9^/L]), characterized by a mature neutrophilia (38.7 × 10^9^/L [RI: 2.4–10.2 × 10^9^/L]) and mild monocytosis (1.3 × 10^9^/L [RI: 0–0.5 × 10^9^/L]) persisted. A polymerase chain reaction antigen receptor rearrangement assay was performed on a sample taken from the cat's mesenteric lymph node, supporting a diagnosis of T‐cell lymphoma.

## 3 Discussion

Hypermagnesemia is uncommon in veterinary species. It may result from excess intake or, more commonly, from decreased renal excretion of magnesium. Few reported cases of oversupplementation or toxicosis exist in veterinary medicine [3]. Legumes and grains are common sources of dietary magnesium, whereas antacids and laxatives are the most readily available nondietary sources [8]. A normal, commercial feline diet was fed regularly to this cat. The most frequently documented causes of hypermagnesemia in cats include chronic kidney disease and obstructive uropathy [1, 2, 4]. This patient presented with no history of kidney disease noted by the referring veterinarian. Patients with normal kidney values may still have a degree of subclinical preexisting kidney disease; therefore, it cannot be definitively dismissed as a contributing factor.

The development of AKI is often multifactorial and is not completely understood in cats [9]. AKI can develop from hemodynamic compromise because the kidneys are susceptible to ischemic injury [9, 10]. We believe that hypermagnesemia resulted in hypotension, subsequently lowering the glomerular filtration rate, which led to an IRIS grade III AKI [7]. Magnesium has never been reported to cause direct nephrotoxicity. AKI results in impaired kidney function and altered homeostasis, which could have potentiated disease progression and contributed to the persistent hypocalcemia [9, 10].

Interestingly, a prevalence study of serum magnesium abnormalities found thoracic neoplasia with concurrent pleural effusion as the lone cause of hypermagnesemia in three nonazotemic cats, one of which was diagnosed with lymphoma [4]. No thoracic neoplasia or pleural effusion was found in the cat in this report; however, lymphoma was diagnosed shortly after discharge from the hospital. Hypermagnesemia is a rarely reported electrolyte abnormality in human patients with cancer [11]. Lymphoma may rarely cause hypermagnesemia through currently undescribed mechanisms in cats. Further study is warranted in this regard.

Vetoquinol^a^, which contains magnesium stearate as an inactive ingredient, was given to the cats in this household. Extensive questioning of the owners revealed no administration of any other medications or supplements containing magnesium. There is limited information available on the specific bioavailability of magnesium stearate. An early study conducted in rats suggested that a dosage of 2500 mg/kg body weight/day did not affect the health status of the subjects [12]. The data currently available in human medicine show dramatic variability in the pharmacokinetics of different magnesium compounds [13]. Based on the available laboratory values and clinical progression, intoxication is suspected as the mechanism of hypermagnesemia in this cat, despite an inability to identify a definitive source.

Magnesium stearate, commonly used in supplements and medications as an anticaking agent, is listed as an inactive ingredient by the manufacturer of vetoquinol [14]. The US Food and Drug Administration (FDA) defines active ingredients as any component of a drug or medication with an intended pharmacological effect, while inactive ingredients are anything that falls outside of that definition [15].

Vetoquinol is not FDA approved as a drug or medication but instead falls into a category of food products “generally recognized as safe” [14]. Therefore, the bioavailability and safety of the various ingredients are not subject to the same standards as medications. Two studies were conducted to investigate the efficacy of vetoquinol; neither study investigated magnesium's bioavailability [16, 17]. Vetoquinol is manufactured at different dosages. Half of a 75‐mg capsule was administered to the other two cats in the household, and this cat may have been ingesting two doses per day, for a total of 75 mg. The amount of magnesium stearate in each capsule is unspecified. Each 75‐mg capsule is specified to weigh 255 mg, meaning it contains 180 mg of inactive ingredients, including the capsule itself. In contrast, the 225‐mg capsule is specified to weigh 250 mg, with only 25 mg of inactive ingredients [18]. The manufacturer was contacted to investigate the composition of the capsules as well as the intention of including magnesium stearate, but no response was received.

Out of concern for the loss of airway protective mechanisms as well as the amount of time elapsed since the suspected intoxication, emesis was not induced when the cat in this report presented [19]. Extracorporeal therapies act to filter toxins from the blood and are commonly used in cases of electrolyte imbalances, especially after the period of effective gastric decontamination has passed [20, 21]. Hemodialysis was discussed but was not elected in this case due to financial limitations. Fluid therapy is often prescribed to dilute concentrations of toxins in the bloodstream, increase renal tubular flow, and reduce reabsorption in the renal tubules [22]. This patient was given a large volume of balanced isotonic crystalloids during the initial resuscitation period (30 mL/kg total boluses in the first 2 h of presentation and maintained on 5 mL/kg/h) with the intention of expanding volume and inducing diuresis. LRS was given to this patient as well. Some preparations of isotonic crystalloids contain magnesium chloride, but the specific product used in this report did not [23].

Furosemide, widely used in small animal veterinary medicine for pulmonary edema and volume overload, was used in the cat in this report to increase renal clearance of magnesium [24, 25]. A loop diuretic, furosemide decreases the activity of the Na─K─2Cl transporters of the ascending limb of the loop of Henle. As previously described, the reabsorption of magnesium is influenced by the electrochemical gradient across the DCT membrane. Inhibition of the transporter should result in diminished capacity of magnesium reabsorption. No veterinary studies have specifically investigated increases in excretion of magnesium with furosemide, but several studies are supportive of a disrupted electrochemical gradient [24, 25]. Most calcium is reabsorbed in the proximal tubule, where furosemide has decreased efficacy [24, 25, 26]. Furosemide may also have contributed to the continued hypocalcemia and increased fluid losses and was discontinued after the first day of hospitalization to minimize these diuretic effects.

Clinical hypocalcemia can present similarly to hypermagnesemia, resulting in weakness, hypotension, and arrhythmias. Calcium gluconate has been used in human medicine as an antagonist for magnesium toxicity [6]. Magnesium and calcium have documented antagonism at the level of the vascular endothelium [27]. In this patient, calcium gluconate was not used as an emergency treatment due to concern for dystrophic calcification. The cat presented with severe hyperphosphatemia concentration (5.7 mmol/L [17.7 mg/dL], RI: 0.9–1.8 mmol/L [2.7–5.7 mg/dL]). In both the human and veterinary literature, a product of calcium multiplied by phosphorous of greater than 65 is reportedly associated with an increased risk of soft tissue mineral deposition [28, 29]. On Day 2 of hospitalization, the cat's hypocalcemia remained, and the concern for potential contribution to the persistent hypotension was increased, so calcium gluconate was initiated by constant rate infusion. Both ionized and total calcium concentrations were measured, and although total calcium decreased, ionized calcium steadily increased throughout hospitalization. The decrease in total calcium was likely secondary to the dilutional effects of fluid therapy and blood loss. Repeated blood sampling could have contributed to hypoalbuminemia and therefore decreased total calcium concentration. Ultimately, calcium supplementation was discontinued before resolution of the hypocalcemia, and clinical improvement continued. Hypocalcemia may have contributed to hypotension to some extent, but the continued normalization of blood pressure while calcium was discontinued suggests that hypocalcemia was no longer a major contributor.

Based on previous studies in people, magnesium is reported to have a dose‐dependent toxicity (Table 3) [5]. In this cat, the peak total magnesium was 7.3 mg/dL, or 6.0 mEq/L (Figure 1). At this concentration, gastrointestinal signs, changes in neurologic status including drowsiness and ataxia, changes in the electrical activity of the heart, and hemodynamic changes including bradycardia and hypotension may be anticipated [6]. Consistent with previous reports, gastrointestinal and cardiovascular effects were seen in this cat.

**TABLE 3:** Expected clinical signs with their associated serum magnesium concentrations [5].

Clinical signs	Serum tMg (mEq/L)
Nausea, vomiting	3.0–3.9
Drowsiness, ataxia	4.0–4.9
Cardiac electrical changes (widened QRS, prolonged PR intervals)	5.0–5.9
Bradycardia, hypotension	6.0–9.9
Obtundation, loss of voluntary motor control	10.0–14.9
Respiratory paralysis	15.0–16.9
Asystole	17.0+

Abbreviation: tMg, total magnesium concentration.

Ionized magnesium is the most clinically relevant measurement of magnesium and traditionally was thought to have a good correlation with total body magnesium due to tight homeostatic regulation [1]. Recent concerns have been raised regarding the correlation between ionized magnesium and total body magnesium concentrations in hospitalized animals [30]. Clinical chemistry panels measuring total magnesium and venous blood gas samples measuring ionized magnesium were used to monitor this cat's magnesium concentration. The values appeared to be correlated. This case report only reflects a single patient, however; therefore, correlation of values in other patients should be interpreted with caution.

Anemia in this cat was not noted on presentation but developed over the first 48 h of hospitalization. The dehydration evident on presentation leads us to believe the cat was severely hemoconcentrated; as blood volume expanded with crystalloids, a normocytic, normochromic, nonregenerative anemia was revealed, persisting for the duration of hospitalization and on follow‐up. The characteristic RBC indices and the acute exacerbation of an inflammatory state are suggestive of anemia of inflammatory disease [31]. Repeated blood sampling may also have contributed to anemia. This diagnosis remained presumptive, and further investigation was not pursued.

In conclusion, this report documents a rare case of hypermagnesemia in a cat. The authors are most suspicious of magnesium toxicosis as the etiology, but underlying kidney disease or neoplasia may have contributed. This level of hypermagnesemia has been documented to result in hypotension and shock, which, combined with other comorbidities, likely resulted in an AKI and persistent refractory hypotension [3, 5]. This report has important limitations, including the unknown source of intoxication and questionable bioavailability of the only known exposure to extradietary magnesium. Additionally, this report lacks generalizability due to a single subject and may be influenced by overinterpretation. Access to over‐the‐counter supplements including magnesium may increase the incidence of recognized intoxication in animals. Identifying magnesium toxicosis in veterinary patients and implementing appropriate treatment protocols is essential. The authors recommend including magnesium assays in bloodwork for all presenting patients to evaluate for dysmagnesemias.

## Author Contributions

**Jackson A. Griffith**: conceptualization, supervision, validation, writing – original draft, writing – review and editing. **Melissa R. Santonocito**: investigation; supervision; writing – review and editing. **Katie D. Mauro**: writing – review and editing.

## Conflicts of Interest

The authors declare no conflicts of interest.

## Endnotes

a Zylkene, Vetquinol USA, 4250 N Sylvania Ave Fort, Worth, TX.

b Metronidazole, Unichem, One Tower Center Boulevard Suite 2200, East Brunswick, NJ.

c Lactated Ringer's Solution, Baxter International, 1 Baxter Pkwy, Deerfield, IL.

d Cerenia (maropitant), Zoetis, 10 Sylvan Way, Parsippany, NJ.

e Convenia (cefovecin), Zoetis, 10 Sylvan Way, Parsippany, NJ.

f Salix (furosemide), Merck, 1 Merck Drive, Whitehouse Station, NJ.

g Calcium Gluconate, Fresenius Kabi, Lake Zurich, IL.

h Recovery Liquid diet, Royal Canin, 500 Fountain Lakes Boulevard Suite 100, Saint Charles, MO.

i Norepinephrine, Fresenius Kabi, Lake Zurich, IL.

j Unasyn (Ampicillin Sulbactam), Pfizer, 235 East 42nd Street, New York, NY.
